# Immunological, biochemical and pathological effects of vitamin C and Arabic gum co-administration on H9N2 avian influenza virus vaccinated and challenged laying Japanese quails

**DOI:** 10.1186/s12917-022-03495-y

**Published:** 2022-11-18

**Authors:** Abdelfattah H. Eladl, Verginia M. Farag, Reham A. El-Shafei, Abeer E. Aziza, Walaa F. Awadin, Nagah Arafat

**Affiliations:** 1 Department of Poultry Diseases, Faculty of Veterinary Medicine, Mansoura University, PO Box: 35516, Mansoura, Egypt; 2 Department of Clinical Pathology, Faculty of Veterinary Medicine, Mansoura University, Mansoura, Egypt; 3 Department of Pharmacology, Faculty of Veterinary Medicine, Mansoura University, Mansoura, Egypt; 4 Department of Nutrition, Faculty of Veterinary Medicine, Mansoura University, Mansoura, Egypt; 5 Department of Pathology, Faculty of Veterinary Medicine, Mansoura University, Mansoura, Egypt

**Keywords:** Vitamin C, Arabic gum, AIV H9N2, Vaccine, Laying quails, Immune response

## Abstract

**Aim:**

This study evaluated the effect of co-administration of vitamin C and Arabic gum (AG) supplements on the response of vaccinated (VAC) and challenged laying Japanese quails with avian influenza virus (AIV) H9N2.

**Materials and methods:**

One hundred and fifty 49-day-old laying Japanese quails were divided into 5 groups (G1-G5): the G1 group was a negative control, G2 group was unvaccinated + H9N2 challenged (Ch), G3 group was unvaccinated + supplements + Ch, G4 group was VAC + Ch, and the G5 group was VAC + supplements + Ch. The supplements **(**vitamin C, 1 g/liter of drinking water and AG, 1% ration) were given for 5 weeks post-vaccination (PV). The birds were injected subcutaneously with an inactivated H9N2 vaccine at 49 days of age. The quails were then challenged intranasally with AIV H9N2 at the 3rd week PV. Blood, tracheal swab and tissue samples were collected at the 1st, 2nd, and 3rd weeks PV, and at different time points post-challenge (PC).

**Results:**

Growth performance, egg production (%), egg and eggshell weights, HI antibody titers, clinical signs, lesions, mortality, virus shedding rates, leukogram, biochemical and immunological parameters and histopathological lesions PC showed significant differences *(P* < 0.05) between the vaccinated-unsupplemented (G4) group and the vaccinated-supplemented (G5) group. G5 showed the highest *(P* < 0.05) growth performance, egg production, HI antibody titers, and heterophil phagocytic activity and the lowest heterophil/lymphocyte (H/L) ratio, mortality, virus shedding rates, creatinine level and histopathological lesion scores in the lungs.

**Conclusion:**

The co-administration of vitamin C and AG for 5 weeks can improve growth performance, egg production and the immune response in vaccinated laying quails challenged with AIV H9N2**.**

**Supplementary Information:**

The online version contains supplementary material available at 10.1186/s12917-022-03495-y.

## Background

Japanese quails (*Coturnix coturnix japonica*) have been reported to be affected by several infectious and non-infectious poultry diseases [1]. Avian influenza virus (AIV) H9N2 is one of the most common poultry diseases, and the epidemics of this virus have been recorded [2]. This virus subtype is a low pathogenic avian influenza virus (LPAIV), and it infects poultry species such as Japanese quails and evidence for antigenic diversity of H9N2 viruses isolated from quails was shown [3]. Peacock et al. [4] reviewed global distribution of H9N2 in poultry including quails. Incomplete protection of the inactivated vaccine against AIV in quails was indicated post-challenge with a field isolate of AIV H9N2, which could infect 30 to 40% of the vaccinated birds. Vaccination of quails with H9N2 induced high antibody titers and the inactivated vaccine did not fully prevent the infection [5].

The presence of dietary vitamin C can enhance protection against Newcastle disease virus (NDV) infection in quails [6]. Moreover, feed intake and egg production were improved with supplementation of vitamin C and vitamin E in quails [7]. Ascorbic acid supplementation either in the diet or in the drinking water improved body weight of broiler chickens [8, 9]. Furthermore, vitamin C has ameliorative effect on renal damage [10, 11].

Arabic gum (AG) contains calcium, magnesium, and potassium and is a branched, neutral or acidic polysaccharide [12]. AG (*Acacia senegal*) has antioxidant properties that can deactivate excited electronic states and scavenge free radicals, and the antioxidant function of its protein fraction (histidine, tyrosine and lysine) acts as an antioxidant molecule [13]. AG is rich in highly soluble fiber and AG can be safely used in broiler diets up to 6% without any adverse effects [14].

Recently, immune responses to different immune stimulants have been used to improve the efficacy of vaccination [15, 16]. As our knowledge, up till now, there are no published articles focused on many factorial influences on the response of vaccinated laying Japanese quails to AIV H9N2 as vitamin C and AG (*Acacia senegal*). Therefore, this study evaluated the effects of vitamin C and AG supplementation on vaccinated quails challenged with chicken field strain of AIV H9N2, assessing (i) growth performance including final body weight (BW) and body weight gain (BWG); (ii) egg production and egg and eggshell weights; (iii) humoral and cellular immune responses (HI test, TLC, differential leukocytic count, H/L ratio, and heterophil phagocytic activity) and liver and kidney function tests (AST, ALT, total protein, albumin, globulin, and creatinine); and (iv) clinical and pathological features (clinical signs, gross lesions, mortality, virus shedding and histopathological lesions).

## Results

### Growth performance, egg production (%), egg and eggshell weights

**Fig. 1:**
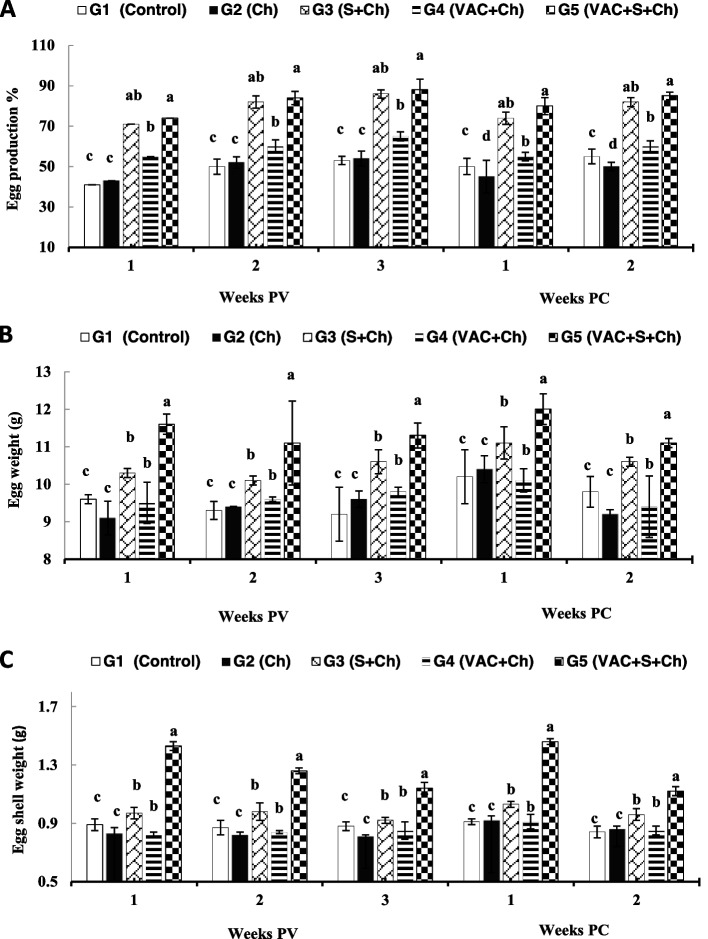
**A** Egg production %, **B** egg weight (g) and **C** egg shell weight (g) results (mean ± SD) of H9N2 vaccinated quails and co-administered vitamin C and Arabic gum (AG). VAC, vaccinated; PV, post-vaccination; PC, post-challenge; G1, negative control; G2, unvaccinated + H9N2 challenged (Ch); G3, unvaccinated + supplemented + Ch; G4, VAC + Ch; and G5, VAC + supplemented + Ch. The supplements (vitamin C, 1 g/l and AG, 1% ration) were given for 5 weeks PV. Means (mean ± SD) with the same letter are not significantly different at *P* < 0.05

In the initial BW, there were no significant differences (*P* > 0.05) between the experimental groups. Quails in G5 had significantly higher final BW at 5 weeks PV (*P* < 0.05) than the other groups (Table 1). BWG displayed a similar result. In this experimental study, G5 showed the highest egg production, followed by G3, G4, and the G2 groups at the 1st, 2nd and 3rd weeks PV and 1st and 2nd weeks PC with AIV H9N2. The egg and eggshell weights followed similar trends (Fig. 1). The birds did not show any eggshell deformities.

**Table 1:** Body weight (BW), body weight gain (BWG), Median clinical signs scores (CSS), HI antibody titers (log2) and mortality % of vaccinated quails and supplemented with vitamin C and Arabic gum (AG) (Mean ± S.D)

Groups	G1	G2	G3	G4	G5
	Control	Ch	S + Ch	VAC + Ch	VAC + S + Ch
**Initial BW (g) (49 days old)**	198.0 ± 1.9^a^	201.6 ± 4.6^a^	199.7 ± 7.5^a^	199.8 ± 3.2^a^	205.1 ± 5.9^a^
**Final BW (g) (5 weeks PV)**	221.9 ± 3.8^c^	212.3 ± 3.2^d^	236.4 ± 7.5^b^	221.5 ± 4.1^c^	245.2 ± 2.3^a^
**BWG (g)**	23.9 ± 0.26^c^	10.7 ± 0.38^d^	36.7 ± 0.29^b^	21.7 ± 0.12^c^	40.1 ± 0.35^a^
**Median clinical signs scores (CSS)**^**a**^**days PC**					
**2nd**	0.0 ± 0.0^e^	0.35 ± 0.21^a^	0.22 ± 0.17^b^	0.15 ± 0.21^c^	0.07 ± 0.01^d^
**5th**	0.0 ± 0.0^e^	0.42 ± 0.21^a^	0.32 ± 0.07^b^	0.26 ± 0.16^c^	0.09 ± 0.02^d^
**9th**	0.0 ± 0.0^c^	0.23 ± 0.81^a^	0.15 ± 0.02^b^	0.12 ± 0.21^b^	0.0 ± 0.0^c^
**14th**	0.0 ± 0.0^c^	0.07 ± 0.01^a^	0.03 ± 0.01^b^	0.02 ± 0.01^b^	0.0 ± 0.0^c^
**HI (Log2) titers**					
**Weeks PV**					
**0**	0.0 ± 0.0	0.0 ± 0.0	0.0 ± 0.0	0.0 ± 0.0	0.0 ± 0.0
**1st**	0.0 ± 0.0^c^	0.0 ± 0.0^c^	0.0 ± 0.0^c^	2.8 ± 0.5^b^	4.2 ± 0.7^a^
**2nd**	0.0 ± 0.0^c^	0.0 ± 0.0^c^	0.0 ± 0.0^c^	3.6 ± 1.4^b^	5.6 ± 0.5^a^
**3rd**	0.0 ± 0.0^c^	0.0 ± 0.0^c^	0.0 ± 0.0^c^	4.3 ± 1.8^b^	6.3 ± 0.9^a^
**Weeks PC**					
**1st**	0.0 ± 0.0^e^	2.6 ± 0.1^d^	3.5 ± 0.4^c^	4.6 ± 0.4^b^	6.6 ± 1.2^a^
**2nd**	0.0 ± 0.0^e^	2.9 ± 0.4^d^	4.1 ± 0.2^c^	5.3 ± 0.6^b^	7.1 ± 0.3^a^
**Mortality % PC with H9N2**	0/30 (0)^d^	3/30 (10)^a^	2/30 (6.7)^b^	1/30 (3.3)^c^	0/30 (0)^d^

Means with the same letter in the same row are not significantly different at *P* < 0.05

*Ch* challenged, *S* supplements, *VAC* vaccinated, *PV* post-vaccination, *PC* post-challenge

^a^CSS (Clinical signs scores) and median clinical signs scores were calculated for each group at 2, 5, 9 and 14 days PC

### Clinical signs, post-mortem lesions and mortality post-challenge with H9N2

The clinical signs were scored from different experimental groups at 2, 5, 9 and 14 days PC and the median clinical signs scores (CSS) was shown in Table 1. Quails in the G2 (Ch) group had significantly higher median CSS than in those of G1 (control) and G5 (VAC + S + Ch) groups at 2, 5, 9 and 14 days PC. The birds challenged with H9N2 showed lack of appetite, depression, ruffled feathers and mild respiratory signs were observed in G2. In contrast, no respiratory signs were observed in the G5. The negative control group showed no mortality. The protection rates against H9N2 correlated positively with the HI test results. The results showed that H9N2 vaccination at 7 weeks of age was effective in quails, as indicated by the HI titres and protection rate (96.7%). The HI titers were significantly higher in G5 than G4. G2 showed mortality (10%), in contrast to the other supplemented and/or vaccinated groups; G3 (6.7%), G4 (3.3%) and there was no mortality (0.0%) in G5 (Table 1). The post-mortem lesions of three dead birds (3/30) in G2 group were observed in different organs including, mild air sacculitis, tracheal and lung congestion and swollen kidney. The H9N2 virus was re-isolated post-challenge from different tissues of the dead quail (trachea and lung) and tracheal swabs in embryonated eggs and gave positive AIV when tested by the haemagglutination test and the detection of H9N2 virus was reconfirmed by real-time RT-PCR.

### HI antibody titers for AIV H9N2

Maternal HI antibody titres for AIV H9N2 were not detected in the experimental quails on day 0 PV (7 weeks old), just before vaccination. G5 showed significantly (*P* < 0.05) higher HI antibody titres against AIV H9N2 than G4 at the 1st, 2nd, and 3rd week PV and at the 1st, and 2nd week PC. No HI antibody titres were found in the control (G1) group at all time points (Table 1).

### Virus shedding rates for AIV H9N2

**Fig. 2:**
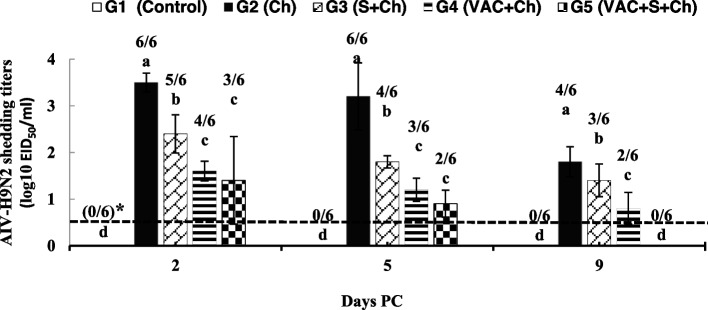
Mean AIV-H9N2 shedding titers (**log10 EID50/ml**) and rates detected in tracheal swabs of different groups (*n*=6). VAC, vaccinated; PV, post-vaccination; PC, post-challenge; G1, negative control; G2, unvaccinated + H9N2 challenged (Ch); G3, unvaccinated + supplemented + Ch; G4, VAC + Ch; and G5, VAC + supplemented + Ch. The supplements (vitamin C, 1 g/l and AG, 1% ration) were given for 5 weeks PV. *Positive shedders ratio = Number of positive quail shedding virus/number of tested quails. Shedding titers (log10 EID50/ml) are expressed as mean ± SD. **The limit of detection was 0.5 log10 EID50/ml**. Means (mean ± SD) with the same letter in the same period are not significantly different at *P* < 0.05

Virus shedding titers and rates in tracheal swabs are shown in Fig. 2. Viral shedding titers and rates for H9N2 were estimated in the swabs from G2 at 2, 5 and 9 days PC, while the shedding was estimated in the swabs from G5 at 2 and 5 days PC, showing that G5 had a short time of shedding. The number of shedders and shedding rates during the 2nd, 5th and 9th day PC were significantly (*P* < 0.05) higher in G2 than in the supplemented groups (G3 and G5) in tracheal swabs. Virus shedding was significantly lower with short period (at 2 and 5 days PC) in G5 than G3 and G4 (at 2, 5 and 9 days PC). The control group (G1) showed no viral shedding at any time point.

### Leukogram, H/L ratio, phagocytic activity and phagocytic index

The leukogram is shown in Table 2 and S1. The TLC, lymphocytes, heterophils and monocytes level showed cleared significant differences between groups while eosinophils and basophils count did not show any significant change. The TLC, lymphocytes and heterophils levels of G4 and G5 were significantly increased at 1st week PV (*P* = 0.0001 of each), in relation to those of others groups. At 1st week PC, the TLC, lymphocytes and heterophils elevated in all experimental groups (G2-G5) in significantly (*P* = 0.0001, *P* = 0.0002, and *P* = 0.0001, respectively) with control one (G1), furthermore, monocytes level of the supplemented groups (G3 and G5) were significantly (*P* = 0.0242) higher than of G1 and G4.

**Table 2:** Leukogram (mean ± S.D) of vaccinated quails, administered vitamin C and Arabic gum (supplements) post-vaccination (PV) and post-challenge (PC)

Groups		G1	G2	G3	G4	G5
		Control	Ch	S + Ch	VAC + Ch	VAC + S + Ch
**1st week PV**	**TLC**	4.51 ± 0.69^c^	4.56 ± 0.63^c^	5.09 ± 0.54^c^	7.93 ± 1.18^b^	9.07 ± 0.82^a^
	**L**	2.75 ± 0.43^c^	2.78 ± 0.40^c^	3.21 ± 0.39^c^	4.20 ± 0.60^b^	5.18 ± 0.63^a^
	**H**	1.68 ± 0.28^b^	1.72 ± 0.24^b^	1.77 ± 0.27^b^	3.53 ± 0.71^a^	3.70 ± 0.36^a^
	**M**	0.04 ± 0.03^a^	0.03 ± 0.04^a^	0.06 ± 0.05^a^	0.09 ± 0.06^a^	0.10 ± 0.09^a^
**2nd week PV**	**TLC**	4.17 ± 0.57^a^	4.23 ± 0.56^a^	5.07 ± 0.83^a^	4.67 ± 0.83^a^	5.02 ± 1.02^a^
	**L**	2.57 ± 0.37^a^	2.56 ± 0.38^a^	3.17 ± 0.52^a^	2.76 ± 0.58^a^	3.15 ± 0.60^a^
	**H**	1.53 ± 0.26^a^	1.61 ± 0.20^a^	1.83 ± 0.36^a^	1.81 ± 0.29^a^	1.83 ± 0.46^a^
	**M**	0.03 ± 0.03^a^	0.04 ± 0.03^a^	0.04 ± 0.04^a^	0.07 ± 0.02^a^	0.03 ± 0.04^a^
**3rd week PV**	**TLC**	4.07 ± 0.52^a^	4.34 ± 0.88^a^	5.00 ± 0.93^a^	4.62 ± 0.86^a^	4.93 ± 0.82^a^
	**L**	2.43 ± 0.31^a^	2.46 ± 0.46^a^	2.96 ± 0.58^a^	2.67 ± 0.39^a^	3.04 ± 0.63^a^
	**H**	1.57 ± 0.24^a^	1.82 ± 0.53^a^	1.89 ± 0.46^a^	1.86 ± 0.54^a^	1.82 ± 0.28^a^
	**M**	0.03 ± 0.04^a^	0.04 ± 0.03^a^	0.05 ± 0.04^a^	0.05 ± 0.05^a^	0.04 ± 0.04^a^
**1st week PC**	**TLC**	4.38 ± 0.94^b^	9.89 ± 1.86^a^	9.53 ± 2.07^a^	9.63 ± 1.95^a^	9.89 ± 2.48^a^
	**L**	2.58 ± 0.58^b^	5.79 ± 1.26^a^	5.82 ± 1.29^a^	5.56 ± 1.13^a^	6.09 ± 1.58^a^
	**H**	1.72 ± 0.38^b^	3.97 ± 0.63^a^	3.44 ± 0.77^a^	3.92 ± 0.91^a^	3.63 ± 0.86^a^
	**M**	0.03 ± 0.04^b^	0.10 ± 0.09^ab^	0.18 ± 0.05^a^	0.09 ± 0.09^ab^	0.13 ± 0.09^a^
**2nd week PC**	**TLC**	4.34 ± 0.78^b^	4.65 ± 0.57^ab^	5.25 ± 0.44^a^	4.50 ± 1.00^ab^	5.36 ± 0.59^a^
	**L**	2.58 ± 0.44^b^	2.77 ± 0.34^ab^	3.14 ± 0.33^a^	2.57 ± 0.60^b^	3.24 ± 0.41^a^
	**H**	1.70 ± 0.33^a^	1.83 ± 0.19^a^	2.02 ± 0.09^a^	1.83 ± 0.40^a^	2.03 ± 0.24^a^
	**M**	0.02 ± 0.03^a^	0.03 ± 0.04^a^	0.06 ± 0.05^a^	0.05 ± 0.05^a^	0.06 ± 0.06^a^

*Ch* challenged, *S* supplements (Arabic gum and vitamin C), *VAC* vaccinated, *TLC* total leukocyte (10^3^/μl) count, *L* lymphocyte (10^3^/μl), *H* heterophil (10^3^/μl), *M*, monocytes (10^3^/μl); G1, negative control; G2, unvaccinated + H9N2 challenged (Ch); G3, unvaccinated + supplemented + Ch; G4, VAC + Ch; and G5, VAC + supplemented + Ch. The supplements (vitamin C, 1 g/l and AG, 1% ration) were given for 5 weeks PV

Means with the same letter (^a-d^) in the same row are not significantly different at *P* < 0.05

**Fig. 3:**
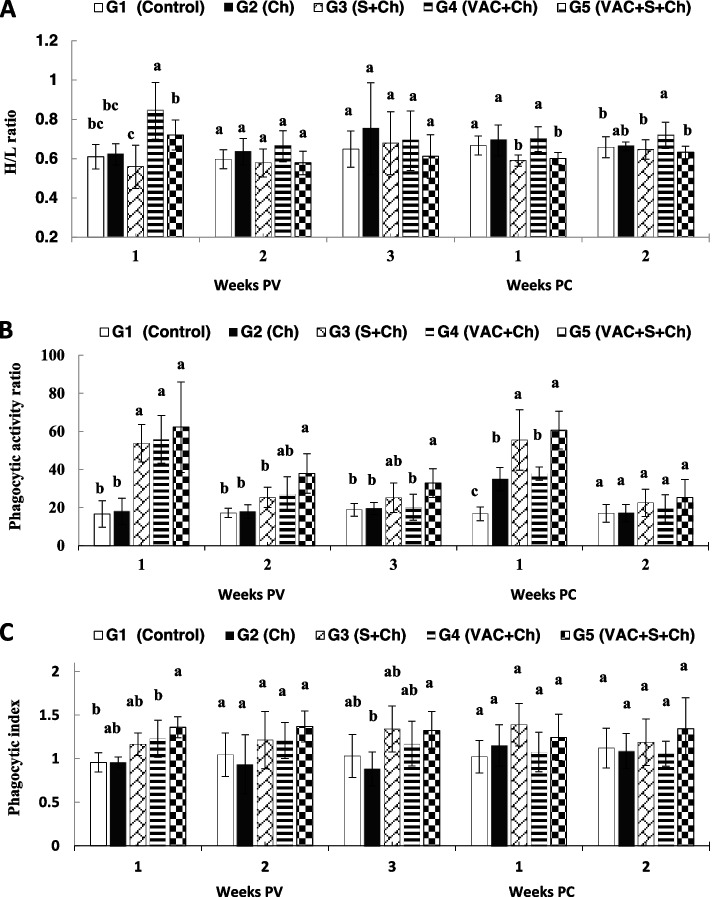
**A** Heterophils lymphocytes ratio (H/L), **B** phagocytic activity ratio and **C** phagocytic index ratio results (mean ± SD) of experimental quails groups; G1, negative control; G2, unvaccinated + H9N2 challenged (Ch); G3, unvaccinated + supplemented + Ch; G4, VAC + Ch; and G5, VAC + supplemented + Ch. The supplements (vitamin C, 1 g/l and AG, 1% ration) were given for 5 weeks PV. PV, post-vaccination; PC, post challenge. Different letters (a–d) on the bars expressed significance level at *P* < 0.05

The H/L ratio of the unsupplemented group G4 had significantly elevated at 1st week PV (*P* = 0.0002) in relation to all others groups, and repeated significant elevation after challenge at 1st week PC (*P* = 0.0021) in relation to G3 and G5 and at 2nd week PC (*P* = 0.0437) than G1, G3 and G5 (Fig. 3A). The phagocytic activity had significantly (*P* < 0.0001) increased in G3-G5 in proportion to G1 and G2 at 1st week PV. Meanwhile that of G5 was significantly increased (*P* = 0.0001) than G1-G3 at 2nd week PV, also it was increased than G1, G2 and G4 at 3rd week PV (*P* = 0.0024) and at 1st week PC (*P* = 0.0001) too (Fig. 3B). As for the phagocytic index (Fig. 3C), only G5 had achieved a significant elevation at 1st week PV in relation to G1 and G4 and at 3rd week PV in relation to G2 (*P* < 0.0001 and *P* = 0.0113, respectively).

### Serum biochemical parameters

At 1st week PV, an elevation of the AST, TP and globulin (*P* = 0.003, *P* = 0.0002, and *P* = 0.0002, respectively) had occurred in the unsupplemented group (G4), however the supplemented one (G5) had elevated TP and globulin than control (G1) and G2 (Table 3). Table S2 demonstrates the biochemical results PC. At 1st week PC, the challenged groups (G2-G4) had elevated liver enzyme AST (*P* = 0.0004) except the supplemented one (G5), which additionally had elevated TP (*P* = 0.0122) and globulin (*P* = 0.0337) content relative to control group (G1). At the same period, all challenged groups G2-G5 suffered from a significant raising creatinine and uric acid (*P* = 0.0013 and *P* = 0.0003 respectively), and those of G2 and G4 continued to rise at 2nd week PC (*P* = 0.0012 and *P* = 0.0001 respectively) with respect to control group.

**Table 3:** Serum biochemical parameters results (mean ± S.D) of vaccinated quails, administered vitamin C and Arabic gum (supplements) post-vaccination (PV)

Groups		G1	G2	G3	G4	G5
		Control	Ch	S + Ch	VAC + Ch	VAC + S + Ch
**1st week PV**	ALT (μ/l)	10.99 ± 3.86^a^	13.11 ± 4.45^a^	13.66 ± 4.11^a^	12.62 ± 4.41^a^	11.66 ± 3.87^a^
	AST (μ/l)	33.13 ± 10.34^b^	30.17 ± 8.57^b^	29.32 ± 9.37^b^	51.75 ± 12.53^a^	30.75 ± 8.82^b^
	TP (g/dl)	2.60 ± 0.36^b^	2.43 ± 0.20^b^	2.96 ± 0.29a^b^	3.50 ± 0.66^a^	3.57 ± 0.50^a^
	Albumin (g/dl)	1.36 ± 0.16^a^	1.48 ± 0.26^a^	1.37 ± 0.18^a^	1.45 ± 0.16^a^	1.45 ± 0.24^a^
	Globulin (g/dl)	1.24 ± 0.34^b^	0.95 ± 0.26^b^	1.59 ± 0.23a^b^	2.05 ± 0.59^a^	2.12 ± 0.56^a^
	Creatinine (mg/dl)	1.00 ± 0.38^a^	1.08 ± 0.20^a^	1.19 ± 0.34^a^	1.10 ± 0.39^a^	1.09 ± 0.32^a^
	Uric acid (mg/dl)	6.23 ± 1.06^a^	7.45 ± 2.23^a^	6.65 ± 1.28^a^	6.80 ± 2.53^a^	8.22 ± 2.76^a^
**2nd week PV**	ALT (μ/l)	12.95 ± 3.30^a^	14.61 ± 4.53^a^	13.66 ± 4.57^a^	12.28 ± 2.88^a^	11.65 ± 4.22^a^
	AST (μ/l)	31.64 ± 10.42^a^	29.89 ± 11.49^a^	30.61 ± 12.82^a^	27.31 ± 11.58^a^	28.28 ± 10.41^a^
	TP (g/dl)	2.35 ± 0.19^a^	2.40 ± 0.20^a^	2.31 ± 0.24^a^	2.63 ± 0.43^a^	2.33 ± 0.19^a^
	Albumin (g/dl)	1.54 ± 0.13^a^	1.48 ± 0.13^a^	1.45 ± 0.20^a^	1.39 ± 0.24^a^	1.46 ± 0.13^a^
	Globulin (g/dl)	0.81 ± 0.18^a^	0.93 ± 0.16^a^	0.86 ± 0.23^a^	1.25 ± 0.64^a^	0.88 ± 0.22^a^
	Creatinine (mg/dl)	1.11 ± 0.42^a^	1.21 ± 0.36^a^	1.10 ± 0.47^a^	1.13 ± 0.39^a^	1.14 ± 0.37^a^
	Uric acid (mg/dl)	7.24 ± 2.70^a^	7.38 ± 2.53^a^	7.09 ± 1.99^a^	7.45 ± 2.91^a^	6.32 ± 0.73^a^
**3rd week PV**	ALT (μ/l)	12.68 ± 3.47^a^	13.24 ± 3.33^a^	11.48 ± 2.57^a^	11.96 ± 2.04^a^	10.84 ± 3.91^a^
	AST (μ/l)	28.04 ± 11.02^a^	31.21 ± 15.93^a^	27.16 ± 10.64^a^	30.74 ± 12.38^a^	27.94 ± 14.63^a^
	TP (g/dl)	2.66 ± 0.39^a^	2.68 ± 0.43^a^	2.63 ± 0.27^a^	2.77 ± 0.37^a^	3.29 ± 0.48^a^
	Albumin (g/dl)	1.70 ± 0.40^a^	1.64 ± 0.49^a^	1.41 ± 0.12^a^	1.49 ± 0.17^a^	1.77 ± 0.43^a^
	Globulin (g/dl)	0.97 ± 0.29^a^	1.05 ± 0.39^a^	1.22 ± 0.28^a^	1.28 ± 0.39^a^	1.53 ± 0.32^a^
	Creatinine (mg/dl)	1.10 ± 0.38^a^	1.34 ± 0.49^a^	1.17 ± 0.40^a^	1.50 ± 0.66^a^	1.13 ± 0.37^a^
	Uric acid (mg/dl)	6.14 ± 2.21^a^	8.73 ± 1.95^a^	7.64 ± 1.58^a^	7.94 ± 3.16^a^	7.33 ± 1.83^a^

*Ch* challenged, *S* supplements (Arabic gum and vitamin C), *VAC* vaccinated, *ALT* alanine aminotransferase, *AST* aspartate aminotransferase, *TP* total protein, G1, G1, negative control; G2, unvaccinated + H9N2 challenged (Ch); G3, unvaccinated + supplemented + Ch; G4, VAC + Ch; and G5, VAC + supplemented + Ch. The supplements (vitamin C, 1 g/l and AG, 1% ration) were given for 5 weeks PV

Means with the same letter (^a-b^) in the same row are not significantly different at *P* < 0.05

### Histopathology

**Fig. 4:**
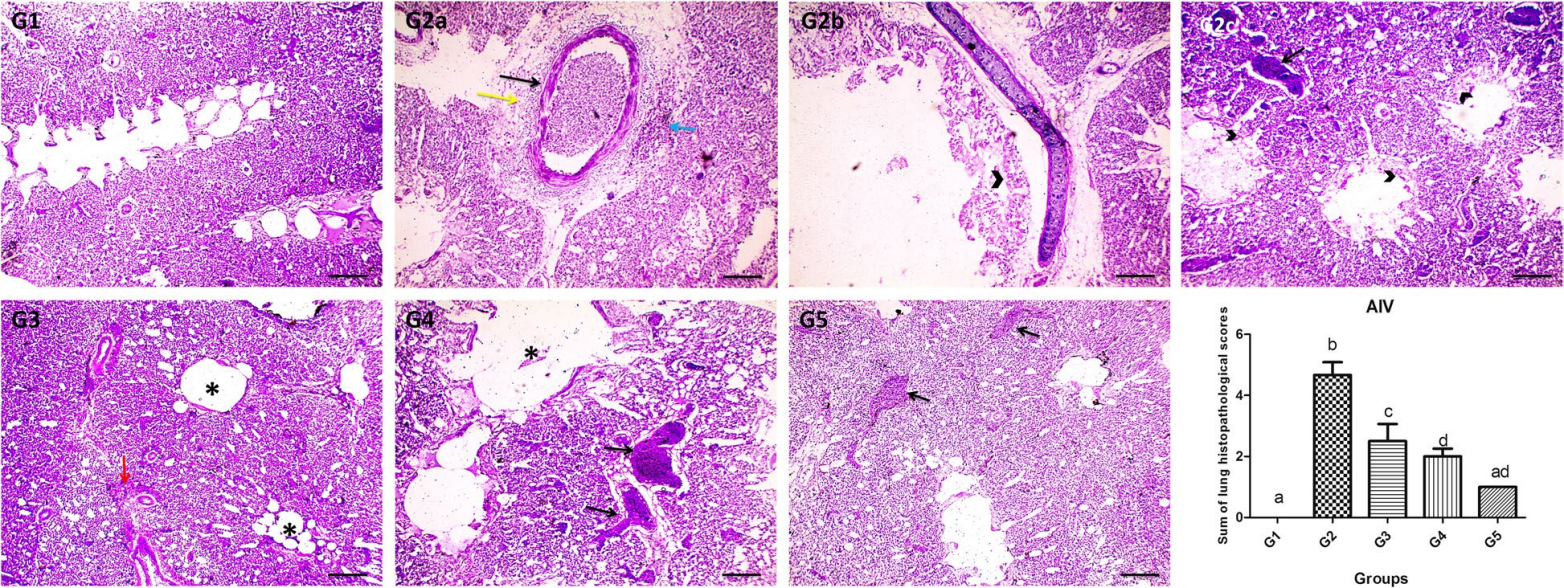
Microscopic pictures of the H&E stained lung sections showing no lesion in G1. Severe congestion (black arrows), perivascular edema (yellow arrow) and heterophils infiltration (blue arrow), necrosis of epithelial cells lining secondary bronchi and parabronchi (arrowheads) were shown in G2 subjected to H9N2 challenge. Perivascular hemorrhage (red arrows) along with narrowing lumen of parabronchi (*) were shown in G3 supplemented with vitamin C + AG and subjected to H9N2 challenge. Mild congestion (black arrows) was shown in G4 vaccinated and subjected to H9N2 infection. Narrowing lumen of few parabronchi (*) and very mild congestion (black arrows) were shown in G5 vaccinated, supplemented with vitamin C + AG and subjected to H9N2 challenge. Scale bar: 100um. Statistical analysis showed significant reduction of total lung lesions scores in treated groups when compared to untreated group G2. Different small alphabetical letters mean significant when *P* < 0.05

Effect of vitamin C and AG supplementation on histopathological lesions in quails vaccinated and challenged with AIV H9N2 was shown in (Fig. 4). Histopathological examination of the lung sections revealed no lesion in G1 but severe congestion, perivascular edema and heterophils infiltration, necrosis of epithelial cells lining secondary bronchi and parabronchi, narrowing lumen of parabronchi in G2. Congestion, perivascular hemorrhage along with narrowing lumen of parabronchi was observed in G3. Mild congestion was observed in G4. Greatly alleviated histological picture was observed in G5**.** Statistical analysis showed significant reduction of total lung lesions scores in supplemented groups (G3 & G5) when compared with unsupplemented group G2 (Fig. 4).

## Discussion

Japanese quails have a role in spreading of AIV especially with inadequate protection of vaccination in this species. Our study evaluated the effect of vitamin C and AG (*Acacia senegal*) co-administration on the response of vaccinated laying Japanese quails (*C. c. japonica*) against challenge with AIV H9N2. In the present study, final BW, and BWG of quails at 5 weeks PV were improved in groups supplemented with vitamin C and AG (G3 and G5). Previous works indicated that the final BW and BWG were significantly higher in chickens [17] and rabbits [18] fed on the AG-supplemented diet. AG produced a significant increase in the BW of laying hens [19]. This enhanced BWG may be because AG is rich in highly soluble fiber. Additionally, Ascorbic acid supplementation either in the diet [8] or in the drinking water [9] improved the BWG of broiler chicks. The supplementation of diet with AG significantly improved the general performance of broilers chicks and economically, AG can be safely used in broiler diets up to 6% without any adverse effects [14]. In the preliminary study, we found that co-administration of vitamin C and AG was superior to each one’s effect in a variety of parameters (data not shown).

In the present study, the vaccinated group supplemented with both vitamin C and AG (G5) showed the highest egg production, followed by the vaccinated only group, both PV and PC with AIV H9N2. The egg and eggshell weights followed similar trends. The vaccinated supplemented group (G5) showed the higher egg and eggshell weights than unvaccinated supplemented group (G3) PV and PC indicating the H9N2 vaccination with supplements has also a beneficial role in the egg and eggshell weights. This result may be related to the immune status and health condition due to vaccination of quails with H9N2 induced high antibody titres [5]. In this study, the AIV H9N2 strain of challenge was isolated from an infected commercial layer flock with severe morbidity and mortality [20]. Nili et al. [21] observed mild clinical signs without mortality in quails infected with AIV H9N2, while feed and water consumption and egg production were decreased. Similarly, Ebrahimi et al. [5] experimentally infected quails with a field isolate of AIV H9N2 (A/Chicken/Iran/339/2002) that was isolated from an infected commercial layer farm that showed reduced egg production and severe morbidity and mortality in the infected quails. In the present study, the birds did not show any eggshell deformities and similar results were previously obtained [21]. Conversely, Qi et al. [22] found that the infected chickens with AIV H9N2 characterized by laying deformed or soft-shelled eggs. A decrease in feed and water consumption is the first clinical sign of LPAIV as a result of a reluctance to move; in addition, reduced egg production can be observed [23]. Abd-Razig et al. [19] found that the addition of 1, 3, 5 and 7% AG to laying hens resulted in a significant increase in egg weight and BW without significant differences in daily egg production. Bardakcioglu et al. [24] also showed that dietary vitamin C could improve egg production and egg and eggshell weights in Japanese quails. Our results showed no clear respiratory signs or nervous system manifestations in the vaccinated and/or supplemented groups (G3-G5) and mortality rates decreased in supplemented groups PC with H9N2. Similarly, the presence of dietary vitamin C can also enhance protection against NDV infection in quails [6].

The results of this study showed that AIV H9N2 vaccination at 7 weeks of age was effective in quails, as indicated by the HI titres and protection rate (96.7%). Ebrahimi et al. [5] showed that vaccination of quails with H9N2 induced high antibody titers; however, a reduction in food and water consumption was evident in the vaccinated-challenged group compared to the unchallenged control group. The authors indicated that the inactivated vaccine did not fully prevent the infection, while protection of birds from clinical signs and decreased viral titers in the lungs after intranasal challenge were shown. In this study, the presence of mortality PC with H9N2 were 10% in unvaccinated quails and 3.3% in vaccinated quails may be due to the pathogenicity of infection and stressed birds during vaccination [15, 25–27]. Meanwhile, in our previous studies, result of mortality post-infection with this H9N2 strain in vaccinated chickens was 0.0% [15] and in unvaccinated quails was 20% [28].

Vitamin C and AG supplementation decreased the number of shedders, shedding titers and rates and period of shedding in tracheal swabs PC with AIV H9N2. This result may be because vitamin C and AG had immune stimulant effects on the immune response of quails vaccinated against H9N2. Wu et al. [29] stated that dietary supplementation of ascorbic acid may protect birds from immunosuppression caused by infectious bursal disease virus vaccination. The immunomodulatory properties of vitamins and medicinal plants were recently studied and nutritive components possess immune stimulant properties without potential toxic effects [15, 30].

Leukogram had marked differences in G3, G4 and G5 indicating the active immune response and cell mobilization from the storage pool of the bone marrow as a result of vaccination. The post challenge changes attributed to the inflammatory response [31]. Moreover, the monocytosis of the supplemented groups (G3 and G5) followed by persistent reactive leukocytosis and lymphocytosis showed the positive immune response of the dietary supplements (vitamin C and AG). Monocytes and lymphocytes increased after vaccination in broilers chickens [32]. The number of monocytes of the present supplemented groups was within normal standard level. Medicinal plants may improve the level of monocytes [33]. Schley and Field [34] reviewed evidence for the immune-stimulating effects of dietary AG. Vitamin C could endogenously formed in poultry, while under vaccination stress the supplementation of this vitamin was needed, it could ameliorating stress due to its antioxidant effect. In addition, vitamin C might helped in T lymphocytes proliferation and differentiation [29, 35].

Concerning the H/L ratio results, it is the best marker to predict stress level in birds. The results were in line with chickens vaccinated and challenged with the AIV H9N2, they had relative heterophilia and lymphopenia [36]. Vitamin C was added in overcoming stress by stimulating lymphocyte elevation and lowering the heterophil count [31]. However, supplementation of vitamin C alone did not influence the immunological state of the broilers but if it had added with another immunostimulant, the response was cleared in case of NDV and IBDV in broilers chicken [37]. However, the AG raised the reactive oxygen species in the granulocytes and led to activate their phagocytic action [29, 35, 37].

Phagocytic activity and index were clearly improved in the supplemented groups at different periods of estimation. Ascorbic acid has a stimulatory effect on leukocyte phagocytic activity and antibody formation, enhances the chemotaxis and phagocytosis of heterophils and regulates B and T cell differentiation and proliferation. The AG, is a powerful immunomodulatory agent, as it activates the extracellular signal-regulated kinases, CD4+ T cells, IL6, IL10, and IL12 [35].

Regarding the biochemical results, vitamin C + AG down-regulated the negative action of the vaccines as represented by lowered AST, uric acid, and creatinine levels and increased levels of total protein, and globulin. Similar results were previously obtained [36]. Aminotransferases (AST and ALT), are a class of enzymes that catalyse the transfer of amino groups between amino acids. There were high in a range of tissues, primarily liver and muscle. In pigeons, AST enzyme was very sensitive for liver cell damage with ethylene glycol. Protein and globulin elevated with dehydration or inflammation. In birds, the excretory functional capability of the proximal tubules of the kidney is reflected in blood uric acid concentration. Uric acid is the avian kidney’s principal nitrogenous waste product, and its level in the blood is a good predictor of renal function [38]. AG is a nephroprotective agent in rats, as it ameliorated oxidative stress and DNA damage due to induced chronic renal failure [39]. The AG contains flavonoids, phenols, coumarin, tannins, polysaccharides and many pharmaceutical and important compounds for improving immunity and health state. It works as an antioxidant, immunomodulator, and cytoprotector [40].

Microscopical examination in this study revealed the protective effects of vitamin C + AG, as indicated by reduced histopathological lesions scores in the lungs of quails challenged with AIV H9N2.These results may be due to the improved immune responses of vaccinated and challenged quails that received the dietary supplements. According to animal studies, vitamin C increased resistance to various viral and bacterial infections. Moreover, many infections, including pneumonia, lead to reduced vitamin C levels in plasma, leukocytes and urine; hence, vitamin C might have a therapeutic effect on pneumonic patients [41]. The study of Oyebanji et al. [42] showed that oral administration of gums with ND vaccination produced protection against the challenge virus, which seems to show preference for the lungs.

Finally, it would be important to study any practical implications and expand on the cost of production and availability of the AG product, if there is going to be a valid market for AG in poultry feed supplementation. Further studies of the effects of different concentrations of AG product on viral infections in chickens are extremely required. Chickens as natural hosts for avian influenza viruses, playing a crucial role in global food security, it might have more veterinary health implications. Thus, future experiments should continue to determine whether there is the same in the effect of vitamin C and AG on the response of vaccinated chickens to H9N2 AIV. This work focused on H9N2 as a low pathogenic avian influenza in quails. In future studies, if the highly pathogenic H5 and H7 AIV in quails or more recent viruses are included, the significance of the work will be raised. It will provide novel insights in the effects of vitamin C and AG on the response of vaccinated laying Japanese quails to AIV. This work showed that supplements could improve immune response in vaccinated laying quails challenged with H9N2 AIV. However, further studies should examine innate and adaptive cellular immune responses in birds and the immune gene expressions should also be examined [43].

## Conclusion

Our results showed significant changes due to vitamin C and AG co-administration in growth performance, egg production (%), egg and eggshell weights, HI antibody titers, clinical signs, gross lesions, mortality rates, virus shedding rates, hematological and biochemical parameters and lung lesions scores. Such a combination can provide protective management for preventing losses of performance in the quail industry due to AIV H9N2 viral infection. In conclusion, the co-administration of vitamin C and AG for 5 weeks can improve growth performance, egg production and the immune response in laying quails vaccinated and challenged with AIV H9N2**.**

## Materials and methods

### Experimental quails

One hundred and fifty 35-day-old laying Japanese quails (originating from the same hatching batch) were obtained from a commercial quail farm in Dakahlia governorate, Egypt, in which there was no history of AIV H9N2 infection. The birds were acclimatized for 2 weeks prior to the experiment. The birds were reared in cages in a hygienic isolated room with a basal balanced diet [44] (Table S3). The birds were not given any medications. Food and water were provided ad libitum during the experiment period (from 49 to 84 days old).

All birds in this study were reared and managed according to the ethical and biosecurity guidelines, and the bird experiment was performed in accordance with all regulations and recommendations of the “Guide for the Care and Use of Laboratory Animals”, which were approved by the Ethical Committee of the Faculty of Veterinary Medicine, Mansoura University. H9 or H5 viruses and antibodies were not detected by isolation in embryonated eggs and the HI testing in the used birds was tested negative right before this experiment [45].

### Supplements, vaccine, antigen, and challenge strain

- Vitamin C: Vitamin C 20% powder contains 200 g of vitamin C per 1 kg and lactose up to 1000 g. It is manufactured by Uni Company, Egypt. The dose was 1 g/litre of drinking water for 5 weeks PV (G3 and G5 groups).
- Arabic gum (*Acacia senegal*): Quails of G3 and G5 groups received a diet that included Arabic gum 1% (Table S3), as previously indicated [19], for 5 weeks PV. Commercial AG (*Acacia senegal*) was obtained from a local market (Mansoura, Egypt) as instant gum powder granule.
- The AIV H9N2 ME Fluvac vaccine contains the strain A/Chicken/Egypt/11490v/NLQP/2011 (group B, G1-like lineage virus). This vaccine is an emulsion (water-in-oil) inactivated vaccine manufactured by Middle East for Veterinary Vaccines Company (MEVAC), Egypt.
- The AIV H9N2 standard diagnostic antigen contains the strain A/Chicken/Egypt/11490v/NLQP/2011 (group B, G1-like lineage virus). It is a low pathogenic AI antigen. This antigen was obtained from MEVAC for the HI test [28].
- The AIV H9N2 strain contains A/chicken/Egypt/Mansoura-36/2015, a low pathogenic AI H9N2 (accession number KX663332) that was kindly provided [20]. This virus is a challenge strain of AIV H9N2 (group B, G1-like lineage virus). Titration of this H9N2 virus was performed in 10-day-old specific pathogen-free embryonated chicken eggs (SPF-ECEs), and the calculation was conducted as previously described [46]. At 70 days of age, the birds (G2- G5) were intranasally challenged with 0.5 ml of 10^6^ EID_50_/bird [28, 47].

### Grouping and experimental design (Table 4)

One hundred and fifty laying Japanese quails were used. At the age of 49 days, the birds were randomly divided into 5 groups (30 birds per group). The G1 group was an unvaccinated negative control, G2 group was unvaccinated + H9N2 challenged (Ch), G3 group was unvaccinated + supplements + Ch, G4 group was VAC + Ch, and the G5 group was VAC + supplements + Ch. G4 and G5 were injected with the AIV H9N2 vaccine subcutaneously at 49 days of age as a common AI vaccination in the field [20, 48]. The birds in G1, G2 and G4 were fed a basal balanced diet. However, the birds of G3 and G5 were supplemented with vitamin C (1 g/litre of drinking water) and AG (1% ration) for 5 weeks PV (from 49th to 84th day of age). At 70 days of age (3rd week PV), G2 to G5 groups were intranasally challenged with the AIV H9N2.

**Table 4:** Grouping and experimental design

Group		G1	G2	G3	G4	G5
		Control	Ch	S + Ch	VAC + Ch	VAC + S + Ch
**Number of quails**		**30**	**30**	**30**	**30**	**30**
**Vaccination (AIV H9N2)**	**49 days of age**	**–**	**–**	**–**	**+**	**+**
**Vitamin C (1 g/liter of drinking water)**	**daily for 5 weeks PV**	**–**	–	**+**	**–**	**+**
**Arabic gum (1% ration)**		**–**	–	**+**	**–**	**+**
**Sampling**	**1st week PV**	• Initial BW at 0 week PV • Serum (*n* = 6) for HI • Egg production %, egg weight and egg shell weight • Leukogram, phagocytic activity and phagocytic index (*n* = 6) • Serum biochemical parameters.				
	**2nd week PV**					
	**3rd week PV**					
**AIV H9N2 challenge** **(*n*** **= 30)**	**3rd week PV**	**–**	**+**	**+**	**+**	**+**
**Sampling**	**1st week PC**	• Final BW and BWG at 5 weeks PV (2 weeks PC) • Serum (*n* = 6) for HI • Clinical signs, mortality %, and post-mortem lesions • Median clinical signs scores (CSS) at 2, 5, 9 and 14 days PC • Egg production %, egg weight and egg shell weight. • Leukogram, phagocytic activity and phagocytic index (*n* = 6) • Serum biochemical parameters • AIV H9N2 tracheal swabs (*n* = 6) shedding rates at 2, 5 and 9 days PC. • Lungs (*n* = 6) for histopathology at 1st week PC.				
	**2nd week PC**					

*Ch* challenged, *S* supplements, *VAC* vaccinated, *AG* Arabic gum, *PV* post-vaccination, *PC* post-challenge, *BW* body weight, *BWG* body weight gain

Six random heparinized blood samples from each group, were collected in the 1st, 2nd and 3rd weeks PV and at 1st and 2nd weeks PC for analysis of leukogram patterns, H/L ratios and phagocytic activities. Six serum samples (from each group) were also collected for the HI test and to determine the biochemical parameters (ALT, AST, total protein, albumin, globulin, creatinine and uric acid). To evaluate the shedding rates, tracheal swabs (*n* = 6, each group) were collected at 2, 5 and 9 days PC. The lungs of the euthanized birds (*n* = 6) by cervical dislocation were collected for histopathological examinations on the 7th day PC.

### Growth performance, egg production, egg weights and eggshell weights

Initial BW was determined at the beginning of the experiment (49 days of age; 0 week PV), while final BW and BWG were calculated at 5 weeks PV. The eggs were collected from 7 to 12 weeks of age (at the 1st, 2nd and 3rd week PV and 1st and 2nd week PC). The eggs were collected daily at the same time, and daily egg numbers were divided by the number of birds for calculation of daily and weekly mean egg production (%). A digital electronic balance was used to measure the egg and eggshell weights [24].

### Clinical signs, post-mortem lesions and mortality rates

The experimental quails were observed twice daily for the recording of clinical signs, post-mortem lesions and mortality rates until 14 days PC. As previously described by Arafat et al. [47], the total clinical signs scores (CSS) per quail and median clinical score for each group were calculated at 2, 5, 9, and 14 days PC. The clinical signs of sneezing, mouth breathing, rales, conjunctivitis, head swelling, nasal and ocular discharge, were scored for each group from 0 to 3; normal (score 0), mild (score 1), moderate (score 2), and severe (score 3) clinical signs.

## Laboratory methods

### Total and differential leukocyte count

The whole heparinized blood samples were used soon for manually counting the TLC. Leukogram assessment was performed [49] using an improved Neubauer haemocytometer and Natt and Herrick solution. Two blood films (for each sample) were performed and stained with Giemsa stain for assessment the differential leukocyte count [50].

### Heterophil phagocytic assay

The *Candida albicans* (*C. albicans*) colonies were kindly obtained from the department of microbiology, Faculty of Medicine, Mansoura University, Egypt. Its suspension were prepared and killed (60 min in a water bath of 56 °C) [51], then the concentration was adjusted (10^7^ cells/ml) using haemocytometer*.* Heterophil phagocytic activity was assayed following Eladl et al. [15] with a little modification. Half ml of the heparinized blood were quickly added to the previously prepared *C. albicans* suspension (50 μl), shaking for 1 min and incubated at 37 °C for 20 min. Then two slides were prepared, and stained with Geimsa stain. The slides were examined under oil immersion (light microscope) lens and 100 heterophils were recorded for the *candida* engulfed. The phagocytic activity was the ratio between the number of phagocytizing heterophils and the number of all examined heterophils. The phagocytic index was calculated as the average number of ingested particles per phagocytizing heterophils [52].

### Serum biochemical analysis

The frozen serum samples were analysed to estimate ALT and AST (Randox Co., UK), creatinine (Human, Germany), uric acid, total protein and albumin (Stanbio, USA) levels following the manufacturer’s instructions. Globulin was calculated as previously described [38].

### HI test

Detection of AIV H9N2 antibody titres using the HI (beta procedure) test was carried out as previously described by Beard [53]. The AIV H9N2 standard antigen was used for the HI test.

### Virus shedding rates in tracheal swabs for AIV H9N2

The tracheal swabs were vortexed with sterile phosphate-buffered saline (PBS) with 1% gentamicin. A QIAamp Viral RNA Mini kit (Qiagen, Germany, GmbH) was used for extraction of viral RNA. PCR reactions were performed in a total volume of 25 μl comprising 7 μl of RNA template, 12.5 μl of 2× QuantiTect Probe RT-PCR Master Mix, 4.125 μl of PCR-grade water, 0.5 μl of each primer (50 pmol), 0.125 μl of probe (30 pmol) and 0.25 μl of QuantiTect RT Mix. The primers and probe sequences used for the real-time RT-PCR were [54]: H9F: 5′-GGAAGAATTAATT ATTATTGGTCGGTAC-3′; H9R: 5′ − CCACCTTTTTCAGTCTGACATT-3′; and H9Probe: [FAM]-AACCAGGCCAGACATTGCGAGTAAGATCC-[TAMRA]. Reverse transcription at 52 °C for 30 min was performed, followed by 40 cycles of denaturation at 94 °C for 15 s, annealing, and extension at 60 °C for 45 s for calculation of a standard curve. The cycle threshold (Ct) values of samples were converted into EID_50_/ml as described previously [55] for the determination of shedding titers. The standard curve was established as described previously [56] for RNA extracted from dilutions of the titrated challenge H9N2 virus. The limit of detection was 0.5 log10 EID_50_/ml. To determine the AIV H9N2 titer, the CT of the collected samples was plotted against the standard curve and the result was represented in log10 EID_50_/ml.

### Histopathology

Lung specimens (*n* = 6 for each group) were fixed in 10% neutral buffered formalin until further processing. Formalin-fixed lung specimens were dehydrated in graded alcohol concentrations, cleared in xylene, and embedded in molten paraffin. Sections of 5 μm were cut, prepared and stained with haematoxylin and eosin as previously described [57]. Examination of stained sections was done using light microscope and pictures were taken with a microscope camera. The degree of histopathological changes included inflammation, congestion, hemorrhage and fibrosis were scored the severity from 0 to 3; no change (score 0), mild (score 1), moderate (score 2), and severe (score 3) blind to the groups [58]. A total score per bird was performed for statistical analysis by summing the scores of observed lesions; minimum total score is zero and maximum total score is 12.

### Statistical analysis

The data were statistically analysed using test of homogeneity of variances (Levene statistic), One-way analysis of variance (ANOVA), then Duncan post hoc test was performed for all estimated parameters in addition to sum of lung lesion scores using Duncan’s multiple range test using SAS 9.2 program for windows. The data were expressed as mean ± SD (*P* < 0.05).

## Supplementary Information

**Additional file 1: Table S1.** Eosinophil and basophil counts (mean ± S.D) of vaccinated quails, administered vitamin C and Arabic gum (supplements) post-vaccination (PV) and post-challenge (PC). **Table S2.** Serum biochemical parameters results (mean ± S.D) of vaccinated quails, administered vitamin C and Arabic gum (supplements) post-challenge (PC). **Table S3.** Ingredients (%) and chemical composition of the control basal diet and Arabic gum 1% diet.

## Data Availability

All data generated or analyzed during this study are included in this article.

## Abbreviations

LPAIV: Low pathogenic avian influenza virus
EID_50_: 50% embryo infective dose
AIV: Avian influenza virus
HI: Haemagglutination inhibition
MEVAC: Middle East for veterinary vaccines company
NLQP: National laboratory for veterinary quality control in poultry production
PV: Post-vaccination
PC: Post-challenge
BW: Body weight
BWG: Body weight gain
TLC: Total leukocyte count
H/L: Heterophil/lymphocyte
g: Gram
VAC: Vaccinated
AG: Arabic gum
ALT: Alanine aminotransferase
AST: Aspartate aminotransferase
