# Supplementary material for: Immunological, biochemical and pathological effects of vitamin C and Arabic gum co-administration on H9N2 avian influenza virus vaccinated and challenged laying Japanese quails
Source: BMC Vet Res. 2022 Nov 18;18:408. doi: 10.1186/s12917-022-03495-y (PMC9673443; doi:10.1186/s12917-022-03495-y)
Supplement: Supplementary file 1 — Additional file 1: Table S1. Eosinophil and basophil counts (mean ± S.D) of vaccinated quails, administered vitamin C and Arabic gum (supplements) post-vaccination (PV) and post-challenge (PC). Table S2. Serum biochemical parameters results (mean ± S.D) of vaccinated quails, administered vitamin C and Arabic gum (supplements) post-challenge (PC). Table S3. Ingredients (%) and chemical composition of the control basal diet and Arabic gum 1% diet. [file 12917_2022_3495_MOESM1_ESM.docx]

**Table S1** Eosinophil and basophil counts (mean ± S.D) of vaccinated quails, administered vitamin C and Arabic gum (supplements) post-vaccination (PV) and post-challenge (PC)

| **Groups** | | **G1** | | | **G2** | **G3** | **G4** | | **G5** | |  |
| --- | --- | --- | --- | --- | --- | --- | --- | --- | --- | --- | --- |
|  |  | **Control** | | | **Ch** | **S + Ch** | **VAC + Ch** | | **VAC + S + Ch** | |  |
| **1^st^**  **week PV** | **E** | | 0.03 ± 0.04^a^ | 0.02 ± 0.03^a^ | | 0.03 ± 0.04^a^ | | 0.09 ± 0.09^a^ | | 0.07 ± 0.06^a^ | |
|  | **B** | | 0.02 ± 0.02^a^ | 0.02 ± 0.02^a^ | | 0.03 ± 0.05^a^ | | 0.02 ± 0.04^a^ | | 0.02 ± 0.04^a^ | |
| **2^n^ week PV** | **E** | | 0.03 ± 0.03^a^ | 0.01 ± 0.02^a^ | | 0.02 ± 0.03^a^ | | 0.02 ± 0.04^a^ | | 0.02 ± 0.02^a^ | |
|  | **B** | | 0.01 ± 0.02^a^ | 0.02 ± 0.02^a^ | | 0.02 ± 0.02^a^ | | 0.0 ± 0.0^a^ | | 0.0 ± 0.0^a^ | |
| **3^rd^ week PV** | **E** | | 0.03 ± 0.03^a^ | 0.02 ± 0.02^a^ | | 0.01 ± 0.02^a^ | | 0.02 ± 0.03^a^ | | 0.02 ± 0.03^a^ | |
|  | **B** | | 0.02 ± 0.02^a^ | 0.01 ± 0.02^a^ | | 0.01 ± 0.02^a^ | | 0.02 ± 0.02^a^ | | 0.02 ± 0.02^a^ | |
| **1^st^ week PC** | **E** | | 0.03 ± 0.03^a^ | 0.04 ± 0.06^a^ | | 0.06 ± 0.08^a^ | | 0.03 ± 0.04^a^ | | 0.04 ± 0.05^a^ | |
|  | **B** | | 0.02 ± 0.03^a^ | 0.0 ± 0.0^a^ | | 0.03 ± 0.05^a^ | | 0.03 ± 0.04^a^ | | 0.0 ± 0.0^a^ | |
| **2^nd^ week PC** | **E** | | 0.02 ± 0.03^a^ | 0.02 ± 0.03^a^ | | 0.04 ± 0.04^a^ | | 0.03 ± 0.04^a^ | | 0.02 ± 0.03^a^ | |
|  | **B** | | 0.02 ± 0.02^a^ | 0.01 ± 0.02^a^ | | 0.0 ± 0.0^a^ | | 0.02 ± 0.03^a^ | | 0.01 ± 0.02^a^ | |

Ch, challenged; S, supplements (Arabic gum and vitamin C); VAC, vaccinated; E, eosinophil (10^3^/µl); B, basophil (10^3^/µl); G1, negative control; G2, unvaccinated + H9N2 challenged (Ch); G3, unvaccinated + supplemented + Ch; G4, VAC + Ch; and G5, VAC + supplemented + Ch. The supplements (vitamin C, 1 g/l and AG, 1% ration) were given for 5 weeks PV. Means with the same letter (^a-d^) in the same row are not significantly different at *P*<0.05.

**Table S2** Serum biochemical parameters results (mean ± S.D) of vaccinated quails, administered vitamin C and Arabic gum (supplements) post-challenge (PC).

| Groups | | G1 | G2 | G3 | G4 | G5 |
| --- | --- | --- | --- | --- | --- | --- |
|  |  | Control | **Ch** | **S + Ch** | **VAC + Ch** | **VAC + S + Ch** |
| **1^st^ week PC** | ALT (µ/l) | 10.71 ± 3.95^a^ | 14.98±4.99^a^ | 13.32±5.08^a^ | 15.08±5.34^a^ | 12.75±3.12^a^ |
|  | AST (µ/l) | 27.31±10.82^b^ | 59.33±16.78^a^ | 50.57±14.43^a^ | 68.25±13.79^a^ | 46.51±11.88^ab^ |
|  | TP (g/dl) | 2.54±0.38^b^ | 3.21±0.43^ab^ | 3.30±0.56^a^ | 2.84±0.39^ab^ | 3.29±0.25^a^ |
|  | Albumin (g/dl) | 1.35±0.33^a^ | 1.46±0.12^a^ | 1.55±0.20^a^ | 1.41±0.23^a^ | 1.42±0.16^a^ |
|  | Globulin (g/dl) | 1.19±0.22^b^ | 1.75±0.46^ab^ | 1.74±0.43^ab^ | 1.44±0.50^ab^ | 1.86±0.26^a^ |
|  | Creatinine (mg/dl) | 1.29±0.40^b^ | 2.09±0.39^a^ | 1.74±0.240.18^a^ | 2.15±0.50^a^ | 1.62±0.11^a^ |
|  | Uric acid (mg/dl) | 8.63±2.50^b^ | 22.54±5.93^a^ | 17.26±3.15^a^ | 20.27±6.96^a^ | 17.99±2.66^a^ |
| **2^nd^ week PC** | ALT (µ/l) | 10.88±3.70^a^ | 14.67 ±3.84^a^ | 12.24±2.89^a^ | 14.12±3.39^a^ | 11.61±2.26^a^ |
|  | AST (µ/l) | 26.98±12.78^a^ | 32.29±9.87^a^ | 28.54±11.50^a^ | 29.11±9.73^a^ | 31.21±10.94^a^ |
|  | TP (g/dl) | 2.54±0.32^a^ | 2.63±0.27^a^ | 2.83±0.51^a^ | 2.41±0.28^a^ | 2.45±0.29^a^ |
|  | Albumin (g/dl) | 1.43±0.10^a^ | 1.49±0.20^a^ | 1.59±0.29^a^ | 1.49±0.11^a^ | 1.40±0.22^a^ |
|  | Globulin (g/dl) | 1.11±0.33^a^ | 1.14±0.40^a^ | 1.24±0.45^a^ | 0.92±0.33^a^ | 1.05±0.22^a^ |
|  | Creatinine (mg/dl) | 1.24±0.26^b^ | 2.11±0.38^a^ | 1.60±0.16^ab^ | 2.10±0.64^a^ | 1.43±0.30^b^ |
|  | Uric acid (mg/dl) | 8.80±2.81^c^ | 20.38±4.51^a^ | 11.82±2.83^c^ | 16.46±2.71^b^ | 12.41±2.63^c^ |

Ch, challenged; S, supplements (Arabic gum and vitamin C); VAC, vaccinated; ALT, alanine aminotransferase; AST, aspartate aminotransferase; TP, total protein; G1, negative control; G2, unvaccinated + H9N2 challenged (Ch); G3, unvaccinated + supplemented + Ch; G4, VAC + Ch; and G5, VAC + supplemented + Ch Means with the same letter (^a-c^) in the same row are not significantly different at *P*<0.05.

**Table S3** Ingredients (%) and chemical composition of the control basal

diet and Arabic gum 1% diet.

| **Arabic gum**  **1% diet** | **Control diet** | **Ingredients** |
| --- | --- | --- |
| 53 | 54 | **Yellow corn%** |
| 36 | 36 | **Soybean meal%** |
| 3 | 3 | **Oil%** |
| 1 | 0.0 | **Arabic gum%** |
| 6.3 | 6.3 | **Limestone%** |
| 0.25 | 0.25 | **Layer premix%** |
| 0.1 | 0.1 | **DL-Methionine%** |
| 0.3 | 0.3 | **Salt** |
| **Chemical composition** | | |
| 19.9 | 19.9 | **CP%** |
| 2900 | 2900 | **ME Kcal/kg** |
| 2.5 | 2.5 | **Ca%** |
| 0.38 | 0.37 | **P%** |
